# Application of Symmetry Adapted Function Method for Three-Dimensional Reconstruction of Octahedral Biological Macromolecules

**DOI:** 10.1155/2010/195274

**Published:** 2010-01-21

**Authors:** Songjun Zeng, Hongrong Liu, Qibin Yang

**Affiliations:** ^1^Institute of Modern Physics, Xiangtan University, Xiangtan 411105, China; ^2^National Laboratory of Biomacromolecules, Institute of Biophysics, Chinese Academy of Sciences, 15 Datun Road, Chaoyang District, Beijing 100101, China

## Abstract

A method for three-dimensional (3D) reconstruction of macromolecule assembles, that is, octahedral symmetrical adapted functions (OSAFs) method, was introduced in this paper and a series of formulations for reconstruction by OSAF method were derived. To verify the feasibility and advantages of the method, two octahedral symmetrical macromolecules, that is, heat shock protein Degp_24_ and the Red-cell L Ferritin, were utilized as examples to implement reconstruction by the OSAF method. The schedule for simulation was designed as follows: 2000 random orientated projections of single particles with predefined Euler angles and centers of origins were generated, then different levels of noises that is signal-to-noise ratio (S/N) = 0.1, 0.5, and 0.8 were added. The structures reconstructed by the OSAF method were in good agreement with the standard models and the relative errors of the structures reconstructed by the OSAF method to standard structures were very little even for high level noise. The facts mentioned above account for that the OSAF method is feasible and efficient approach to reconstruct structures of macromolecules and have ability to suppress the influence of noise.

## 1. Introduction

The determination of three-dimensional (3D) structures of macromolecular assemblies plays a key role in understanding their functions and properties. In the course of structure reconstruction of single particles during the last several decades, the Cryo-electron microscopy (referred to as “CryoEM”) has been successively used to solve 3D structures up to subnanometer resolution [1–6], even near-atomic resolution, such as the 3.8 Å resolution structures of the CPV [7] and rotavirus [8], the 4.2 Å GroEL structure [9], and the 4.5 Å Epsilon 15 bacteriophage structure [10]. Such large assemblies often are either too large or too heterogeneous to be able to study by the conventionalX-ray crystallography and nuclear magnetic resonance (NMR) [11, 12]. It is considered that the CryoEM is an indispensable approach for determining the 3D structures of macromolecular complexes. And many different software packages have been developed in the world wide laboratories for 3D reconstructions, such as EMAN [13], FREALIGN [14] using the direct Fourier inversion method, MRC [15] using the Fourier-Bessel synthesis method and spherical symmetry-adapted functions (SAFs) method [16, 17]. The SAF method was first realized to be a more efficient method indicated by Crowther in their pioneer paper [18] three decades ago. Provencher and Vogel implemented 3D reconstruction by the SAF method taking both simulated structures and biological objects as samples [19, 20]. Zheng et al. used an icosahedral SAF (ISAF) method to determine the structures of viruses from solution X-ray scattering data [21]. Navaza [16] systematically developed formulations for 3D reconstruction of icosahedral viruses including ab initio determination of origins and orientations of particles and interpolation of data in the reciprocal space by the ISAF method. Recently our group [17] reconstructed icosahedral symmetry biological objects (HBV, etc.) by icosahedral SAF approach which showed that SAF method is an efficient approach to reduce the influence of noise and achieve high resolution because of its ability of completely utilizing symmetry operation of the object being studied. Up to date, all the SAF method is only used in the reconstruction of icosahedral symmetry macromolecular. Due to a variety of symmetry of the macromolecular existing in nature except icosahedral symmetry, such as octahedral (small heat shock protein hsp16.5) [22, 23], heat shock protein Degp_24_ [24], Hepatitis B small surface antigen particles (HbsAg) [25] tetrahedral (small heat shock protein ACR1 [26]), dihedral (auxilin-bound clathrin coat [27] catalase, ribulose bisphosphate, glutamine synthetase, carboxylase/oxygenase) and so forth, we would like to extend SAF method to reconstruct the objects having any other symmetry, for example, octahedral, tetrahedral, dihedral symmetries. 

In this paper, we would like to concentrate our attention to the octahedral symmetry-adapted function (OSAF) method. And a series of formulations for 3D reconstruction of octahedral symmetry macromolecules had been derived. To verify the feasibility and the advantages of this approach, two octahedral symmetrical simulated data such as heat shock protein Degp_24_ (3Cs0.pdb) [24] and the Red-Cell L Ferritin (1rcc.pdb) [28] downloaded from protein data bank (PDB) have been reconstructed by the OSAF method at high resolution. The results demonstrate that the OSAF method can retrieve the 3D structures of the octahedral symmetrical objects at high resolution and effectively suppress the influence of noise.

## 2. Principle of the OSAF Method

For readily understanding the OSAF method for 3D reconstruction, it is necessary to describe the principle of the OSAF method briefly.

### 2.1. Octahedral Symmetry-Adapted Functions (OSAFs)

Due to SAFs being the linear combinations of the spherical harmonics *Y*_*l*,*m*_(*θ*, *φ*) [29], the major problem is how to find the coefficients *B*_*l*,*m*_^*μ*^ of the OSAF (see expression (1)). According to the conventional definition, we choose the *X*, *Y*, *Z* axes of the Cartesian coordinates system along three 4 fold axes of an octahedral symmetry group, respectively, and the relationship between the Cartesian and spherical pole coordinates is as convention. Consequently, the OSAF can be written as follows:

(1) $$O_{l,\mu }\left(\theta ,\varphi \right)=\overset{l}{\underset{m=-l,m=0\;(\mathrm{mod}\;4)}{\sum }}B_{l,m}^{\mu }Y_{l,m}\left(\theta ,\varphi \right),$$

where the *O*_*l*, *μ*_(*θ*, *φ*) represents the OSAFs, the *Y*_*l*,*m*_(*θ*, *φ*) denotes the normalized spherical harmonics, and the *B*_*l*,*m*_^*μ*^ is the combination coefficient with *m* = 0 (mod 4) required by the *Z* axis being along a 4-fold axis, *μ*(*l*) is multiplicity of a given order *l* of an OSAF. All the *μ*(*l*) for *l* ≤ 11 are listed in the Table 1, when *l* ≥ 12, the values of the *μ*(*l*) can be obtained by

(2) $$\mu \left(l\right)=\mu \left[\mathrm{mod}\;\left(\frac{l}{12}\right)\right]+\mathrm{int}\;\left(\frac{l}{12}\right),$$

where int (*l*/12) is the integer part of the (*l*/12) and mod (*l*/12) the reminder of *l* divided by 12.

To calculate OSAFs, at first, one should calculate the normalized spherical harmonics by the formula

(3) $$Y_{l}^{m}\left(\theta ,\varphi \right)=\sqrt{\left\{\frac{\left(2l+1\right)\left(l-\left|m\right|\right)!}{4\pi \left(l+\left|m\right|\right)!}\right\}}P_{l}^{m}\left(\cos\;\;\theta \right)\exp\;\;\left(im\varphi \right),$$

where the *P*_*l*_^*m*^(cos *θ*) is the Legendre-associated polynomial. Then, it is essential to solve the combination coefficients in (1) for getting the OSAFs. Up to now, there are many methods to obtain the high-order OSAFs, such as the general methods [30], Algebraic method [31], and the recursive approach [32]. But we prefer the recursive method since it is less sensitive to the computation errors and more stable for achieving the higher order OSAF as pointed out by Schmidt [32]

(4) $$B_{l_{1}+l_{2},M}^{\mu }=\gamma \left(l_{1},l_{2},l_{1}+l_{2}\right)\times \overset{l}{\underset{m=-l}{\sum }}\sqrt{\frac{\left(2l_{1}\right)!\left(2l_{2}\right)!\left(l_{1}+l_{2}+M\right)!\left(l_{1}+l_{2}-M\right)!}{\left[2\left(l_{1}+l_{2}\right)+1\right]!\left(l_{1}+m\right)!\left(l_{1}-m\right)!\left(l_{2}+m-M\right)!\left(l_{2}-m+M\right)!}}\times B_{l_{1},m}^{\mu_{1}}B_{l_{2},M-m}^{\mu_{2}},$$

where *γ*(*l*_1_, *l*_2_, *l*_1_ + *l*_2_) is normalized constant.

According to (4), any high-order OSAF can be generated only by using three lower order seed OSAFs with *l* = 4, 6, 9. The coefficients *B*_*l*,*m*_^*μ*^ of the seed OSAFs are listed in the Table 2.

For example,

(5) $$\begin{array}{cc} O_{4,1}\left(\theta ,\varphi \right) & =\frac{\left(\sqrt{21}\cdot Y_{4}^{0}\left(\theta ,\varphi \right)+\sqrt{15/2}\cdot \left(Y_{4}^{-4}\left(\theta ,\varphi \right)+Y_{4}^{4}\left(\theta ,\varphi \right)\right)\right)}{6},\\ O_{9,1}\left(\theta ,\varphi \right) & =\frac{\left(\sqrt{51}\cdot \left(Y_{9}^{4}\left(\theta ,\varphi \right)-Y_{9}^{-4}\left(\theta ,\varphi \right)\right)-\sqrt{21}\cdot \left(Y_{9}^{8}\left(\theta ,\varphi \right)-Y_{9}^{-8}\left(\theta ,\varphi \right)\right)\right)}{12}. \end{array}$$

Due to the properties of the normalized spherical harmonic functions, the *Y*_*l*,*m*_(*θ*, *φ*) and *B*_*l*,*m*_^*μ*^ satisfy the following relationships:

(6) $$\begin{array}{c} B_{l,m}^{\mu }={\left(-1\right)}^{l-m}B_{l,-m}^{\mu },\\ Y_{l,m}^{*}\left(\theta ,\varphi \right)={\left(-1\right)}^{m}Y_{l,-m}\left(\theta ,\varphi \right). \end{array}$$

So that

(7) $$\begin{array}{cc} & B_{l,m}^{\mu }Y_{l,m}\left(\theta ,\varphi \right)+B_{l,-m}^{\mu }Y_{l,-m}\left(\theta ,\varphi \right),\\ & \;\;=B_{l,m}^{\mu }\left[Y_{l,m}\left(\theta ,\varphi \right)+{\left(-1\right)}^{l}Y_{l,m}^{*}\left(\theta ,\varphi \right)\right]. \end{array}$$

(8) $$\begin{array}{cc} & \text{When}\;\;l\;\;\text{is}\;\;\text{even},\\ & \;m\neq 0\to O_{l,\mu }^{c}\left(\theta ,\varphi \right)=2B_{l,m}^{\mu }P_{l}^{m}\left(\cos\;\;\theta \right)\cos\;\;m\varphi ,\\ & \;m=0\to O_{l,\mu }^{c}\left(\theta ,\varphi \right)=B_{l,m}^{\mu }P_{l}^{m}\left(\cos\;\;\theta \right)\cos\;\;m\varphi .\\ & \text{When}\;\;l\;\;\text{is}\;\;\text{odd},\\ & \;m\neq 0\to O_{l,\mu }^{s}\left(\theta ,\varphi \right)=2B_{l,m}^{\mu }P_{l}^{m}\left(\cos\;\;\theta \right)\sin\;m\varphi ,\\ & \;m=0\to O_{l,\mu }^{s}\left(\theta ,\varphi \right)=0, \end{array}$$

where the asterisk∗ denotes complex conjugation, *O*_*l*, *μ*_^*c*^(*θ*, *φ*) and *O*_*l*, *μ*_^*s*^(*θ*, *φ*) denote the real and the imaginary parts of the OSAF, respectively.

According to (8) one may use the *O*_*l*, *μ*_^*c*^(*θ*, *φ*) to fit the real part of structure factor and *O*_*l*, *μ*_^*s*^(*θ*, *φ*) the imaginary part of structure factor of biological objects with octahedral symmetry in reciprocal space. All the *O*_*l*, *μ*_^*c*^(*θ*, *φ*) and *O*_*l*, *μ*_^*s*^(*θ*, *φ*) of OSAFs with *l* ≤ 12 are listed in Table 3. The first column gives the orders of OSAF, the second gives the multiplicity *μ*(*l*) of OSAF, *c* and *s* in the third denotes the *O*_*l*, *μ*_^*c*^(*θ*, *φ*) and *O*_*l*, *μ*_^*s*^(*θ*, *φ*), respectively, the final column presents the combination coefficients *B*_*l*,*m*_^*μ*^ of the OSAF, and the (0), (4), (8), and (12) mean *m* = 0,4, 8, and 12, respectively. For example, according to (8), we can gain the OSAFs of *l* = 6,9:

(9) $$\begin{array}{cc} O_{6,1}^{c}\left(\theta ,\varphi \right) & =0.35355339059\times P_{6}^{0}\left(\cos\;\;\theta \right)\cos\;\;\left(0\right)\\ & \;-0.66143782777\times \left(2.0\times P_{6}^{4}\left(\cos\;\theta \right)\cos\;\left(4\varphi \right)\right),\\ O_{9,1}^{s}\left(\theta ,\varphi \right) & =0.59511903571\times \left(2.0\times P_{9}^{4}\left(\cos\;\;\theta \right)\sin\;\left(4\varphi \right)\right)\\ & \;-0.38188130791\times \left(2.0\times P_{9}^{8}\left(\cos\;\theta \right)\sin\;\left(8\varphi \right)\right). \end{array}$$

**Figure 1:**
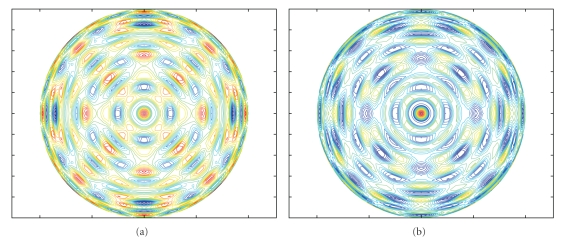
Density contour maps of the OSAFs looking down along a fourfold axis. (a) *l* = 40, *μ* = 1, (b) *l* = 40, *μ* = 2.

Figures 1(a) and 1(b) show the density contour lines of the OSAFs looking down along a fourfold axis with *l* = 40, *μ* = 1 and 2, respectively. We can get all the higher order of OSAFs up to 1000 via (4).

It should be pointed out that the OSAFs (*O*_*l*, *μ*_(Θ, Φ)) are the orthogonal complete basis if the multiplicity *μ* is taken all the values, then any function *F*(*R*, Θ, Φ) with octahedral symmetry can be represented in terms of the linear combination of *O*_*l*, *μ*_(Θ, Φ).

### 2.2. Reconstruction Principle

It is well known that the structure of macromolecular complexes can be described as its potential functions which are determined by Fourier inversion transformation of the structure factors *F*(**R**), and its expression in spherical coordinates is

(10) $$\rho \left(r\right)=\int F\left(R\right)\exp\;\;\left(-2\pi iR\cdot r\right)R^{2}\sin\;\Theta \;dR\;d\Theta \;d\Phi ,$$

where **r** and **R** denote the vectors in real and Fourier spaces, respectively.

In contrast to Crowther's Fourier-Bessel method [33], we utilize OSAF to express *F*(*R*)

(11) $$F\left(R\right)=F\left(R,\Theta ,\Phi \right)=\overset{\infty }{\underset{l=0}{\sum }}\;\overset{n_{l}}{\underset{\mu =1}{\sum }}f_{l,\mu }\left(R\right)O_{l,\mu }\left(\Theta ,\Phi \right),$$

where *f*_*l*, *μ*_(*R*) is the fitting coefficient of the OSAF, and its value depends on the Fourier radius *R* of a spherical shell and *l*, *μ*; *n*_*l*_ is the maximum multiplicity of a given order *l*.

According to (8), (11) can be further expressed as the following two parts, that is, real and imaginary parts,

(12) $$\begin{array}{cc} F_{r}\left(R,\Theta ,\Phi \right) & =\overset{\infty }{\underset{l=0\;(\mathrm{mod})\;2}{\sum }}\;\;\overset{n_{L}}{\underset{\mu =1}{\sum }}f_{l\text{even},\mu }\left(R\right)O_{l,\mu }^{c}\left(\Theta ,\Phi \right),\\ iF_{i}\left(R,\Theta ,\Phi \right) & =i\overset{\infty }{\underset{l=1\;(\mathrm{mod})\;2}{\sum }}\;\;\overset{n_{l}}{\underset{\mu =1}{\sum }}f_{l\text{odd},\mu }\left(R\right)O_{l,\mu }^{s}\left(\Theta ,\Phi \right), \end{array}$$

where *F*_*r*_(*R*, Θ, Φ) and *F*_*i*_(*R*, Θ, Φ) denote the real and imaginary parts of *F*(*R*, Θ, Φ), respectively.

Substituting (12) into (10), one can finally obtain

(13) $$\begin{array}{cc} & \rho \left(r,\theta ,\varphi \right)\\ & =4\pi [\overset{\infty }{\underset{l=0\mathrm{mod}\;2}{\sum }}{\left(-i\right)}^{l}\overset{n_{L}}{\underset{\mu =1}{\sum }}\left(\int_{0}^{\infty }f_{l\text{even},\mu }\left(R\right)j_{l}\left(2\pi Rr\right)R^{2}dR\right)\\ & \;\;\;\times O_{l,\mu }\left(\theta ,\varphi \right)+\overset{\infty }{\underset{l=1\mathrm{mod}\;2}{\sum }}{\left(-i\right)}^{l}i\\ & \;\;\;\times \overset{n_{L}}{\underset{\mu =1}{\sum }}\left(\int_{0}^{\infty }f_{l\text{odd},\mu }\left(R\right)j_{l}\left(2\pi Rr\right)R^{2}dR\right)O_{l,\mu }\left(\theta ,\varphi \right)], \end{array}$$

where *j*_*l*_(2*π* *R* *r*) labels the spherical Bessel functions, and its recurrence relationship can be seen in reference [34].

The reconstruction by OSAF can be carried out in the following procedure.

1. Calculate the OSAFs by (1) up to the required order.
2. Construct two linear equation groups with experimental determined structure factors *F*_*r*_(*R*, Θ, Φ) and *F*_*i*_(*R*, Θ, Φ) in the reciprocal space according to (12).
3. Find the fitting coefficients *f*_*l*even, *μ*_(*R*) and *f*_*l*odd, *μ*_(*R*) by solving the above two linear equation groups by means of the least square method.
4. Determine 3D structures of octahedral symmetrical objects according to (13).

## 3. Implementation of Reconstruction by the OSAF Method

To verify the feasibility and advantages of the OSAF method for reconstruction of macromolecules with octahedral symmetry, two biological objects with octahedral symmetry, heat shock protein Degp_24_ and the Red-cell L Ferritin, were taken as examples. The atomic structures were downloaded from PDB (3cs0.pdb and 1rcc.pdb). The both 3D structures with 4.0 Å resolution were generated as standard structure models (SSMs) by the EMAN's pdb2mrc procedure. Then two thousand random projections of these two proteins with predefined orientations and centers were created using real-space projection. Then random noise was added to each projection at 3 different signal to noise ratios (*S*/*N*), that is, 0.1, 0.5., and 0.8 for 3D reconstruction according to the definition of *S*/*N* which is described as below

(14) $$\frac{S}{N}=\frac{\bar{\text{signal}}}{\bar{\text{noise}}},$$

where $\bar{\text{signal}}$ is the average value of the signal, and $\bar{\text{noise}}$ the average value of the noise.

**Figure 2:**
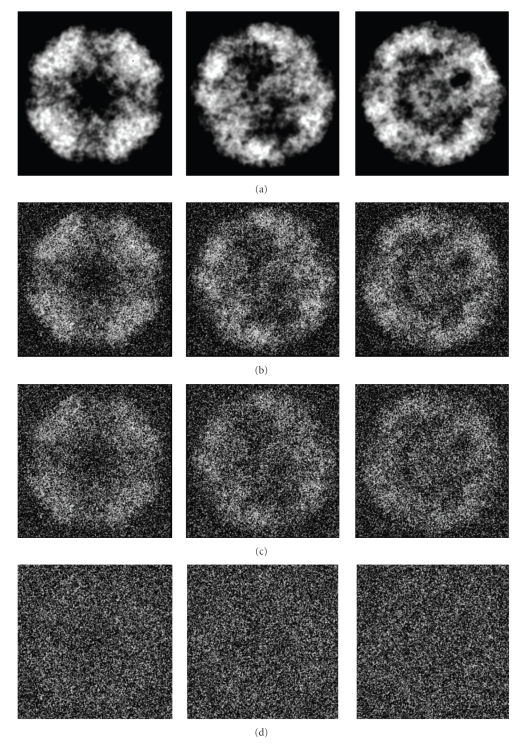
Three representative projection images of the DegP_24_ model generated from PDB data. (a) Without noise, (b) with *S*/*N* = 0.8, (c) with *S*/*N* = 0.5, (d) with *S*/*N* = 0.1.

**Figure 3:**
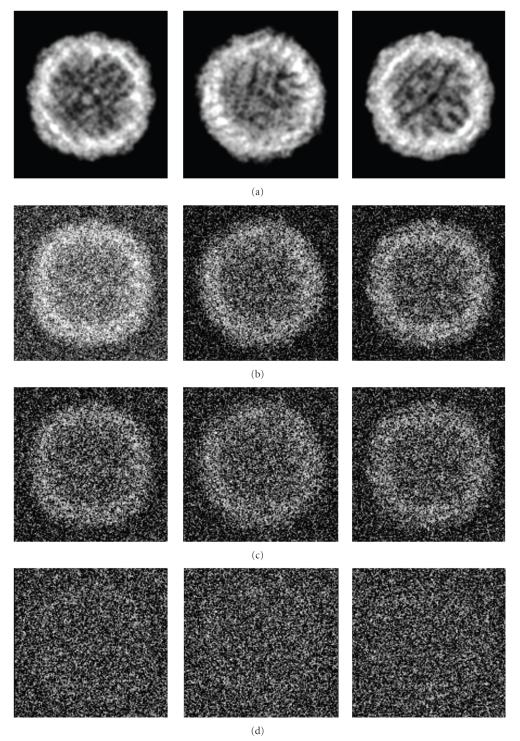
Three representative projection images of the erythrocyte L Ferritin model generated from PDB data. (a) Without noise, (b) with *S*/*N* = 0.8, (c) with *S*/*N* = 0.5, (d) with *S*/*N* = 0.1.

**Figure 4:**
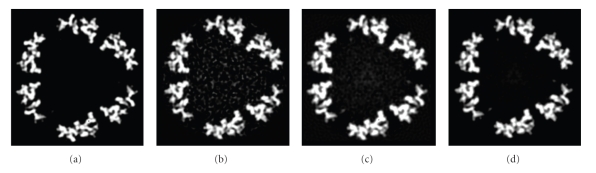
Maps of two-dimensional sections perpendicular to *Z* axis with *Z* = 0 of the DegP_24_ model and reconstruction results viewing along the 3-fold axis by the OSAF method: (a) the standard model, (b) the reconstructed results with *S*/*N* = 0.1, (c) *S*/*N* = 0.5, (d) *S*/*N* = 0.8.

**Figure 5:**
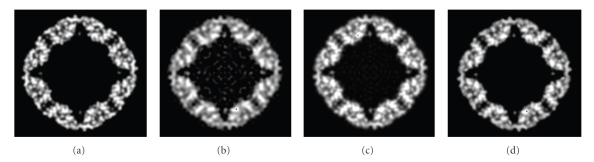
Maps of two-dimensional sections perpendicular to *Z* axis with *Z* = 0 of the erythrocyte L Ferritin model and reconstruction results viewing along the 3-fold axis by the DSAF method: (a) the standard model, (b) the reconstructed results with *S*/*N* = 0.1, (c) *S*/*N* = 0.5, (d) *S*/*N* = 0.8.

**Figure 6:**
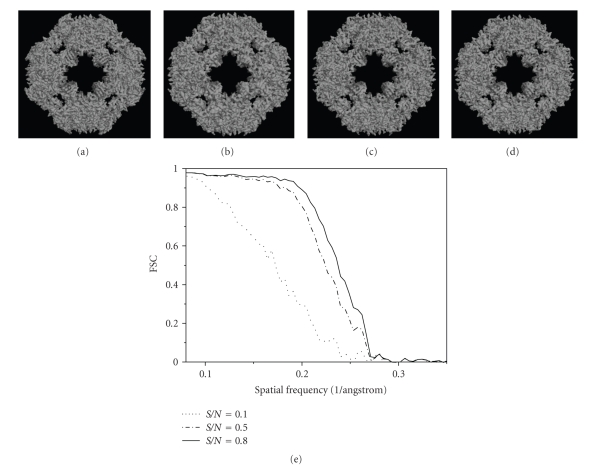
3D structures reconstructed by the OSAF method viewing along the 4-fold axis of DegP_24_ protein with *S*/*N* = 0.1, 0.5, and 0.8. (a) 3D structures of SSM, (b) the result for *S*/*N* = 0.1, (c) *S*/*N* = 0.5, (d) *S*/*N* = 0.8, (e) the FSC curves for different noise levels.

**Figure 7:**
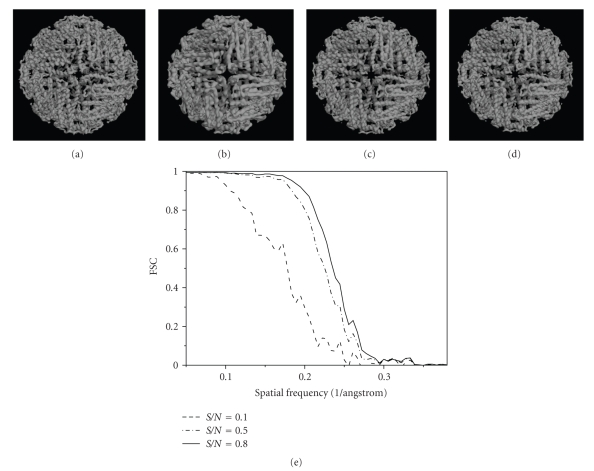
3D structure viewing along the 4-fold axis of erythrocyte L Ferritin model with *S*/*N* = 0.1, 0.5, and 0.8. (a) The standard 3D structures, (b) the result for *S*/*N* = 0.1, (c) *S*/*N* = 0.5, (d) *S*/*N* = 0.8, (e) the FSC curves for different noise levels.

**Figure 8:**
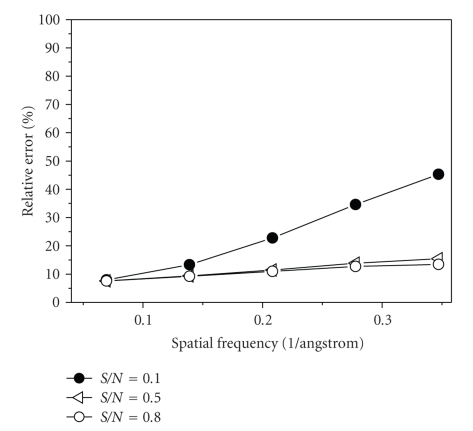
The relative error between the erythrocyte L Ferritin model and the results reconstructed by the OSAF method.

Finally, 3D structures of two models by making use of all the 2000 projections (*S*/*N* = 0.1, 0.5, and 0.8) with predefined random Euler angles and center parameters were reconstructed at high resolutions by the OSAF approach. Figures 2 and 3 show typical projections of the DegP_24_ and the Red-cell L Ferritin proteins with *S*/*N* = 0.1,0.5,0.8 and without noise, respectively. Figures 4 and 5 show the two-dimensional section maps perpendicular to *Z* axis with *z* = zero viewing along the 3-fold axis of DegP_24_ and 4-fold axis of Red-cell L Ferritin proteins, respectively. From the two-dimensional section maps, one may find that some noises still present in the final structures. Usually a perfect reconstruction is impossible in the case of added noise. However it can be seen intuitively from Figures 4 and 5 that the structures reconstructed by the OSAF method are in good agreement with the SSMs even with heavy noise *S*/*N* = 0.1. Figures 6 and 7 show the comparison of 3D reconstructed structures of above two proteins in high resolution, respectively, by the OSAF method. Although there is no obvious discrepancy between the 3D density map reconstructed by the OSAF method for *S*/*N* = 0.1,0.5,0.8, a few differences still can be identified. For quantitative comparison, the Fourier shell correlations (FSCs) [35] listed in Figures 6(e) and 7(e) were calculated, respectively. According to the FSC = 0.5 criterion, the nominal resolution of reconstructed results of the DegP_24_ protein is approximately equal to 5.8 Å, 4.6 Å, and 4.3 Å and those of Red-cell L Ferritin protein, are approximately equal to 5.6 Å, 4.7 Å, and 4.2 Å with *S*/*N* = 0.1,0.5, and 0.8, respectively. It can be seen from Figures 6 and 7 that the reconstructed structures with *S*/*N* = 0.8 have the approximately nominal resolutions as the SSMs and the attainable resolutions decrease with the increase of the added noises (*S*/*N* = 0.5 and *S*/*N* = 0.1). Naturally, more particles are needed to achieve high resolution for very low *S*/*N* = 0.1. To show the quality of the reconstructed structures by the OSAF method for different *S*/*N*s in the real space quantitatively, the relative errors (REs) of the reconstructed 3D structures of Red-cell L Ferritin model with added different noise levels such as *S*/*N* = 0.1,0.5, and 0.8 deviated to the SSMs have been calculated and presented in Figure 8. The formula for calculating RE is described as follows [36]:

(15) $$\text{RE}=\frac{\sum \left|\rho_{o}-\rho_{r}\right|}{\sum \left|\rho_{o}\right|}\times 100\text{\%},$$

where RE denotes the Relative Errors between the SSMs and reconstructed structures, *ρ*_*o*_ represents potential of SSM and *ρ*_*r*_ is that of the normalized structure reconstructed by OSAF method. From Figure 8, one may find that with the increase of the Fourier frequency, the RE increases gradually. Furthermore it is apparent that in the case of the low Fourier frequency with nominal resolution of 14.4 Å, the relative errors listed in Table 4 show that REs keep almost constant for the different *S*/*N*s, which means that the reconstructed structures are hardly influenced by added noise even for *S*/*N* = 0.1. As the structure reconstructed by the OSAF method is of very low RE, that is to say, the structure reconstructed by the OSAF method is very close to the real structure. As the added noises increase, the RE increases. The fact mentioned above implies that the OSAF method is feasible and efficient approach to reconstruct structures of macromolecules and can suppress the influence of noise since the OSAF method can completely utilize 24 symmetry operations of the octahedral symmetry. To achieve advantage of symmetry operation, one should use symmetry adapted function (SAF), for example, icosahedral, octahedral, tetrahedral, dihedral SAF, all these functions have ability to suppress influence of noise in different extent depending on the number of symmetry operation. The icosahedron have 60 symmetry operations which have the strongest ability which is verified by our former paper [17], but other SAFs have certain ability to suppress the influence of noise.

Since the *S*/*N* of raw CryoEM data is very low, one may need a large number of particles to reconstruct a 3D structure to achieve high resolution. Therefore the program for dealing with these huge particles for 3D reconstruction is very time consumption. It is essential to reduce the computation time of the 3D reconstruction from huge particles at high resolution. To achieve fast computation, we managed to carry out the reconstruction in an asymmetrical unit of an octahedral symmetry, and therefore the calculation was speeded up 24 times so that the reconstruction can be performed with a PC computer which will be described in another paper in detail. Table 5 shows the algorithm time by the OSAF method. All the above tests were carried out at a general PC flat with the Pentium D 3.2 GHz CPU and the 2 G RAM. From the Table 5 one can see that the OSAF method is very fast even for high resolution reconstruction.

## 4. Conclusions

1. A set of formulations for 3D reconstruction of macromolecular assemblies with octahedral symmetry by the OSAF method has been established.
2. The OSAF method is feasible and efficiently suppresses the influence of the noise because of its sufficiently utilizing the symmetry of the objects.
3. The calculation can be greatly speed up by dealing with the reconstruction in an asymmetrical unit of the octahedral symmetry group.

It should be pointed out that in the simulation, one may use projections with predetermined centers and orientations to reconstruct structures; however in practice, one should reconstruct based on experimented measured data with unknown centers and orientations. In this case, one should first determine the center and orientation of a projection. At this stage, we did not write a program to determine the center and orientation by the OSAF method itself yet, So far we should use the other program such as EMAN [13], FREALIGN [14], and so forth, to determine the orientation and center parameters of a particle, then adopt the OSAF method for reconstruction. The orientation definition in our OSAF method is the Z-X-Z convention by “clockwise rotation” which is identical to the EMAN's program and is different from Z-Y-Z convention such as FREALIGN's. The relationship of orientation definition between OSAF method and Z-Y-Z convention can be described as follows which is in the same way as EMAN:

(16) $$\begin{array}{c} \phi_{z1}=\phi_{z}+\frac{\pi }{2},\\ \theta_{x}=\theta_{y},\\ \psi_{z1}=\psi_{z}-\frac{\pi }{2}, \end{array}$$

where *ϕ*_*z*1_, *θ*_*x*_, *ψ*_*z*1_ is the Z-X-Z convention adopted in our OSAF method and EMAN, *ϕ*_*z*_, *θ*_*y*_, *ψ*_*z*_ is the Z-Y-Z convention adopted in SPIDER, IMAGIC, MRC, and FREALIGN.

The OSAF method for reconstruction is just at beginning stage, there is a plenty of space for optimizing the program. We believe that this method has a prospective future. A reconstruction with experimental data is proceeding based on the principle mentioned above and will be reported later on.

## Figures and Tables

**Table 1:** The multiplicity *μ*(*l*) of the OSAF with *l* ≤ 11.

*l*	0	1	2	3	4	5	6	7	8	9	10	11
*μ*(*l*)	1	0	0	0	1	0	1	0	1	1	1	0

**Table 2:** The coefficients of the OSAF for *l* = 4, 6, 9.

*l*	*m* = 0	*m* = 4	*m* = 8	Normalized constant
4	$\sqrt{21}$	$\sqrt{15/2}$	*N*/*A*	6
6	$\sqrt{2}$	$\sqrt{7}$	*N*/*A*	4
9	*N*/*A*	$\sqrt{51}$	$-\sqrt{21}$	12

**Table 3:** The OSAFs of all *l* ≤ 12.

*l*	*μ*(*l*)		The combination coefficients of real OSAFs
0	1	*c*	1(0)
4	1	*c*	0.76376261583(0) + 0.45643546459(4)
6	1	*c*	0.35355339059(0) − 0.66143782777(4)
8	1	*c*	0.71807033082(0) + 0.27003085064(4) + 0.41142538219(8)
9	1	*s*	0.59511903571(4) − 0.38188130791(8)
10	1	*c*	0.41142536788(0) − 0.41457793389(4) − 0.49344671132(8)
12	1	*c*	0.65528231041(0) + 0.34173950429(4) + 0.41076683405(8) + 0.40844750473(12)
12	2	*c*	0.23308636320(0) − 0.29796028267(4) + 0.61972174691(8) + 0.0(12)

**Table 4:** The Relative Errors with different *S*/*N* at low Fourier frequency (*R* = 1/14.4 Å^−1^).

Fourier radius	*S*/*N*	Relative errors
1/14.4 Å^−1^	*S*/*N* = 0.1	7.96%
1/14.4 Å^−1^	*S*/*N* = 0.2	7.62%
1/14.4 Å^−1^	*S*/*N* = 0.5	7.60%
1/14.4 Å^−1^	*S*/*N* = 0.8	7.59%

**Table 5:** The algorithm time of the OSAF method for reconstructing DegP_24_ model at low and high resolution (S means second).

Particle numbers	Time (*R* = 1/18.4 Å^−1^)	Time (*R* = 1/6.8 Å^−1^)
500	17 (S)	165 (S)
2000	37 (S)	186 (S)
5000	88 (S)	230 (S)
